# Catheter ablation of tachyarrhythmias in children: state of the art and future directions

**DOI:** 10.1007/s00399-025-01102-9

**Published:** 2025-09-15

**Authors:** Ferran Roses-Noguer, Joachim Hebe

**Affiliations:** 1 Paediatric Cardiology Department, Vall Hebron University Hospital, Passeig de la Vall d’Hebron, 119-129, 08035 Barcelona, Spain; 2 Royal Brompton Hospital, Guy’s and St Thomas Foundation Trust, London, UK; 3 Autonomous University of Barcelona, Barcelona, Spain; 4 Universitat Central de Catalunya, Vic, Barcelona Spain; 5 Center for Electrophysiology, Bremen, Germany

**Keywords:** Cardiac ablation, Paediatrics, Congenital heart diseases, Intracardiac echocardiography, Digital twins, Katheterablation, Pädiatrie, Angeborene Herzfehler, Intrakardiale Echokardiographie, Digitale Zwillinge

## Abstract

**Introduction:**

Paediatric catheter ablation procedures have traditionally relied heavily on fluoroscopic guidance, exposing children to ionizing radiation and the associated long-term cancer risks ranging from 0.4 to 6.0% of total lifetime cancer risk. This necessitates technological innovations to minimize radiation dependency while maintaining therapeutic effectiveness.

**Objectives:**

To review current state-of-the-art technologies and emerging innovations in paediatric catheter ablation.

**Materials and methods:**

This review examines advanced three-dimensional (3D) mapping algorithms, including low voltage bridge mapping, late annotation electrograms, open window mapping, and omnipolar technology for substrate characterization. Intracardiac echocardiography and digital twin frameworks utilizing cardiac magnetic resonance imaging, computed tomography, and electrocardiogram data for personalized procedural planning are also analyzed.

**Results:**

The implementation of advanced 3D mapping systems demonstrates a marked reduction in radiation exposure while optimizing procedural outcomes across structurally normal and complex congenital heart disease patients. Intracardiac echocardiography provides high-resolution real-time imaging, eliminating fluoroscopic dependence and enabling dynamic clinical decision-making during complex procedures. Focal pulsed field ablation may have a role in paediatric ablations, as it potentially reduces the risk of damaging the conduction tissue and other nearby structures.

**Conclusions:**

The integration of advanced imaging technologies represents transformative progress in paediatric electrophysiology, enabling safer, more precise interventions with significantly reduced radiation exposure. These innovations establish new paradigms for personalized paediatric cardiac care, promising improved long-term outcomes for vulnerable populations requiring catheter ablation procedures.

## Abstract (de)

**Einführung:**

Katheterablationen bei Kindern werden traditionell überwiegend unter röntgengestützter Orientierung durchgeführt, wodurch Kinder ionisierender Strahlung ausgesetzt werden. Dies ist mit einem erhöhten Risiko einer Krebserkrankung von 0,4 bis 6,0 % für die gesamte Lebenszeit verbunden. Daher sind dringlich technologische Innovationen erforderlich, um die Strahlenbelastung zu minimieren bei gleichzeitiger Erhaltung der therapeutischen Wirksamkeit.

**Ziele:**

Der aktuelle Stand der Technik und neue Innovationen in der pädiatrischen Katheterablation werden zusammengefasst.

**Material und Methoden:**

Diese Übersicht analysiert fortschrittliche 3‑dimensionale (3-D) Mapping-Algorithmen wie „low voltage bridge mapping“, Late-annotation-Elektrogramme, „open window mapping“ und Omnipolar-mapping-Technologie zur Substratcharakterisierung. Die intrakardiale Echokardiographie sowie „digital twin frameworks“, in denen kardiale Magnetresonanztomographie‑, Computertomographie- und Elektrokardiogramm-Daten für eine personalisierte Verfahrensplanung Nutzung finden, werden ebenfalls untersucht.

**Ergebnisse:**

Die Implementierung fortschrittlicher 3‑D-Mapping-Systeme zeigt eine deutliche Reduktion der Strahlenexposition bei gleichzeitiger Optimierung der Behandlungsergebnisse sowohl bei Patienten mit strukturell normalem Herzen als auch bei Patienten mit komplexen angeborenen Herzfehlern. Die intrakardiale Echokardiographie ermöglicht eine hochauflösende Echtzeitbildgebung, hebt die Abhängigkeit von der Fluoroskopie auf und erlaubt dynamische klinische Entscheidungen während komplexer Eingriffe. Die fokale Pulsed-field-Ablation könnte bei pädiatrischen Ablationen eine wichtige Rolle spielen, da sie möglicherweise das Risiko einer Schädigung des Reizleitungssystems und anderer benachbarter Strukturen reduziert.

**Schlussfolgerungen:**

Die Integration fortschrittlicher Bildgebungstechnologien stellt einen transformativen Fortschritt in der pädiatrischen Elektrophysiologie dar. Sie ermöglicht sicherere und präzisere Eingriffe bei deutlich reduzierter Strahlenbelastung. Diese Innovationen schaffen neue Paradigmen für die personalisierte pädiatrische Herzmedizin und versprechen verbesserte Langzeitergebnisse für gefährdete Patientengruppen, die Katheterablationen benötigen.

## Introduction

The landscape of paediatric catheter ablation has seen remarkable technological advancements designed to improve outcomes in treating tachyarrhythmias. A critical concern in this field is the radiation exposure that paediatric patients face during cardiac intervention procedures. This highlights the pressing need for innovations that can mitigate such risks while ensuring effective treatment.

Modern tools, such as advanced three-dimensional (3D) mapping systems and intracardiac echocardiography, have emerged to enhance anatomical visualization and procedural safety. The adoption of new algorithms developed by novel 3D mapping systems, such as omnipolar mapping (OPM), low voltage bridge (LVB), late annotation electrograms (LAE), and open window mapping (OWM) along with novel energies such as focal pulsed field ablation (PFA) catheters, embodies a shift towards precision in interventions, tailoring approaches to individual patient needs. Furthermore, non-invasive strategies involving digital twins are gaining traction, promising to provide deeper insights into cardiac structures while significantly reducing radiation dependency. The following sections will delve into these advances, addressing their implications for personalized paediatric care.

## Reducing radiation exposure in paediatric catheter ablations

In the realm of paediatric catheter ablation, minimizing radiation exposure has become a paramount concern due to the unique sensitivity of children to radiation effects compared to adults. The long-term risks associated with radiation exposure during cardiac catheterization procedures can be significant, with children treated using fluoroscopy-guided technology facing a measurable risk of increased cancer incidence later in life. This risk poses a long-term cancer risk of 0.4–6.0% of children’s total lifetime cancer risk, mainly attributable to increased risks of lung and breast cancers [1]. This is influenced by a variety of factors, including the type of procedure, the patient’s age and sex, and the individual radiation dose received. The lower the age, the higher the risk, with girls in particular having a higher risk compared to teenagers or adults. This necessitates a proactive approach to mitigating radiation exposure within paediatric populations undergoing catheter ablation.

The integration of advanced imaging fluoroscopy technologies that can reduce radiation, and especially the use of 3D mapping systems for anatomical guidance, have been widely adopted by paediatric electrophysiologists around the world. These tools are used not only for ablations in complex heart diseases but also for procedures in patients with structurally normal hearts, such as in the case of atrioventricular reentrant tachycardia (AVRT), atrioventricular nodal re-entrant tachycardia (AVNRT), or focal atrial tachycardia (FAT). The constant development in signal interpretation and automatic annotation using the latest versions of the 3D mapping systems have led to the introduction of new mapping algorithms that facilitate substrate characterization and electrical activation, leading to enhanced precision in catheter placement. The emerging data suggest that such curated mapping strategies not only optimize procedural outcomes but also lead to a marked reduction in radiation exposure.

## Advances in 3D mapping systems

Recent advances in electroanatomical 3D mapping systems have revolutionized the management of paediatric arrhythmias [2, 3], allowing a precise anatomical and electrical understanding of each patient’s arrhythmic substrate and significantly improving efficacy and safety, particularly in marcroreentrant atrial and ventricular tachycardia (VT)-related post-surgical isthmuses [3, 4]. Furthermore, the use of electroanatomical 3D mapping systems has been adopted worldwide to guide cardiac ablations of other substrates such as AVNRT, AVRT, or FAT in structurally normal hearts. Particularly the introduction of new algorithms, such as LVB, LAE, OWM, and OPM, to delineate anatomical and electrical substrates has been described as useful in paediatric patients.

**Fig. 1:**
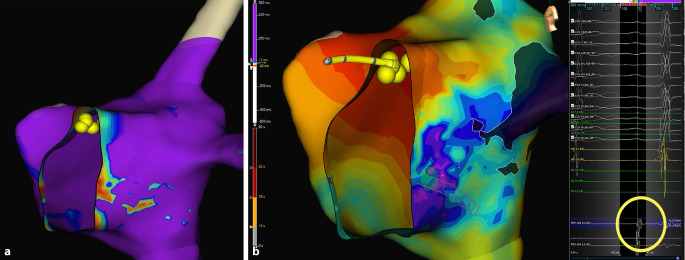
**a** Lateral view of a 13-year-old boy with atrioventricular nodal re-entry tachycardia. Right atrium voltage map created with Ensite X demonstrating a low voltage bridge area corresponding to the posteroseptal region. **b** Ensite X local activation time map with last deflection annotation to identify the region of latest activation in the slow pathway area (red = early, purple = late)

LVB mapping is a novel method for visualizing the slow pathway electrical substrate using adjusted voltage maps in 3D electroanatomical mapping systems. This technology can be used with either high density electrode catheters or conventional ablation catheters to delineate the low voltage potentials observed in the slow pathway region. This strategy has proved effective in the treatment of AVNRT in children using cryoablation (CRYO) [5, 6], allowing the use of both radiofrequency (RF) and ablation energies (Fig. 1a). Other authors have embraced this option and added a second step to the algorithm, consisting of the additional identification of fractionated electrograms (EGMs) with late activation that correspond to the terminal input of the slow conducting AV nodal pathway into the AVNRT circuit. The combination of voltage maps and automatic late annotation EGMs, using either the last deflection with Ensite systems (Abbott, Abbott Park, Illinois, USA) or last activation mapping (LAM) with Carto (Irvine, California, USA), is a highly precise tool to guide AVNRT ablations (Fig. 1b).

**Fig. 2:**
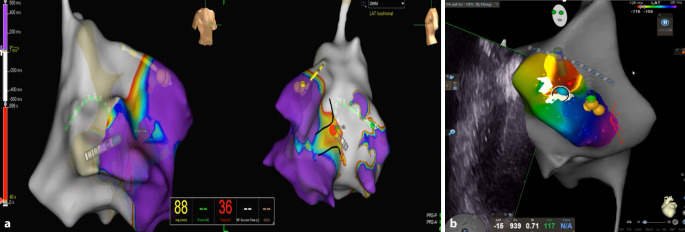
**a** An 11-year-old girl with a right mid-septal accessory pathway mapped with open window mapping (OWM) using Ensite X performed with a conventional non-irrigated tip ablation catheter. **b** A 10-year-old boy with a right anterior accessory pathway mapped with an OWM and CartoSound system using a non-irrigated Navistar ablation catheter

Another important achievement to emerge in recent years is the use of OWM for the identification of accessory pathways [7]. In OWM, both atrial and ventricular EGM signals are included in the window of interest (WOI), and there is automatic algorithm-driven detection of local signals demonstrating the maximum change in voltage over time (dV/dt). Therefore, there is no need to identify atrial, ventricular, or pathway EGMs, which can be difficult to visualize in some cases, particularly with widely spaced bipolar mapping ablation catheters, and may depend on the operator [8]. OMW was initially described using high density mapping electrodes, but it can also be easily carried out with conventional ablation catheters when carefully mapping around the suspected accessory pathways, both towards the atrium and ventricle, thereby enabling visualization of the activation direction through the atrioventricular (AV) annulus (Fig. 2). This technology can be especially useful in complex cases such as Ebstein’s anomaly, where EGM identification can be extremely challenging due to, for example, longer courses of accessory pathways or their wider insertions, particularly at atrialized portions of the right ventricle.

**Fig. 3:**
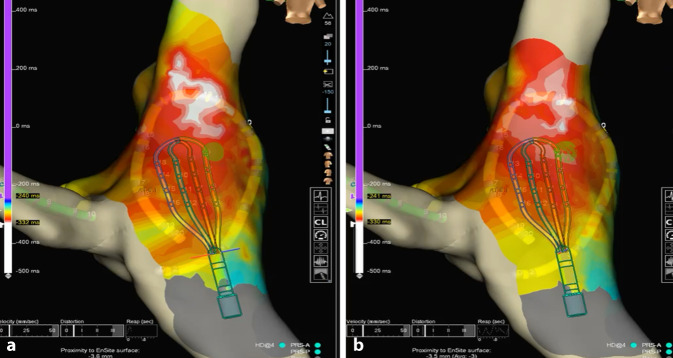
A 14-year-old boy with right superior and lateral focal atrial tachycardia (FAT). Local activation time (LAT) map with Ensite X and high-density grid electrode is performed comparing bipolar vs omnipolar mapping (OPM). **a** The bipolar LAT map shows a broad area of early signals, whereas (**b**) with OPM shows a much more precise early region (white = earliest site of activation)

Finally, the introduction of high-density multipolar mapping electrodes has allowed the introduction of omnipolar technology. This technology obtains EGMs from a set of three orthogonal poles that provide true maximum voltage of local signals, activation direction, and wave speed independent of catheter/wavefront orientation [9]. This technique enhances the detection of arrhythmogenic substrates through its automated analysis capabilities, allowing detailed insights that may otherwise remain obscured and enabling more precise interventions during catheter ablation [10, 11]. This novel technology is particularly useful in patients with complex macroreentrant circuits associated with complex congenital heart diseases and/or complex postoperative anatomy [11], but it has also been used for AVNRT in patients with structurally normal hearts ([12], Fig. 3).

Evaluating the effectiveness and safety of these advanced 3D mapping systems involves analyzing specific metrics that contribute to the overall procedural success. These metrics encompass acute success rates, recurrence rates, and complication rates post-ablation. For instance, the recurrence rate of atrial arrhythmias following procedures guided by LVB and OPM is notably low, reinforcing their clinical utility. Studies indicate that recurrence is generally linked to arrhythmogenic substrate complexity rather than the ablation technique itself. These results affirm that with enhanced mapping technologies, clinicians can achieve higher success rates and minimize long-term complications for paediatric patients.

## The role of intracardiac echocardiography

The integration of intracardiac echocardiography (ICE) into paediatric catheter ablation procedures has marked a significant advance in enhancing the safety and efficacy of these interventions. Traditionally, catheter ablation in the paediatric population has predominantly relied on fluoroscopy, exposing young patients to ionizing radiation. In this context, ICE provides a compelling alternative by offering high spatial and temporal resolution to identify cardiac structures in real time; it is also the only reliable surrogate that offers the opportunity to minimalize, and often eliminate, the need for fluoroscopic guidance. Moreover, ICE has been shown to reduce procedure time without compromising the effectiveness of the procedures [13]. The use of ICE has been rapidly embraced in the world of adult electrophysiology [14, 15], as it is particularly useful in mapping certain anatomic structures that are not well visualized by fluoroscopy—particularly the aortic cusps and intracavitary structures such as the papillary muscles, false chordae, right ventricular moderator band, and left ventricular summit. For the latter structures, ICE has proven itself in the ablation of cardiac arrhythmias [16].

**Fig. 4:**
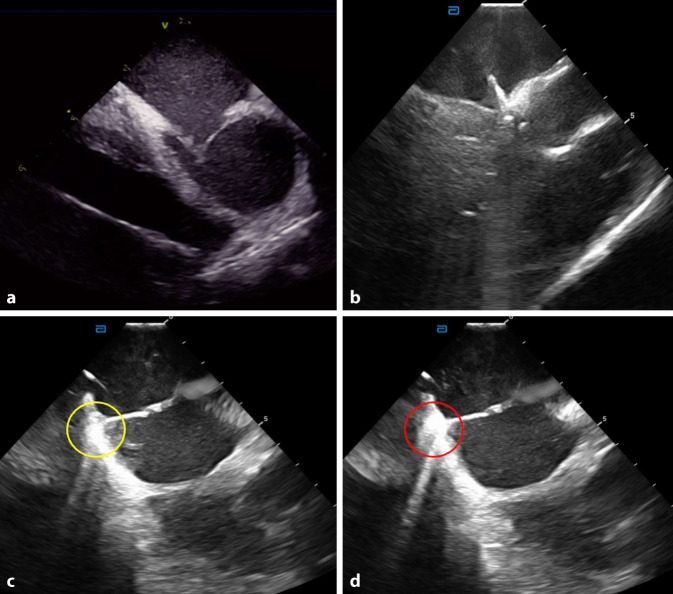
**a** A 6-year-old girl with left-sided atrioventricular tachycardia. Transeptal puncture performed with intracardiac echocardiography. The tip of the Brockenbrough needle is tenting against a floppy intra-atrial septum only 4 mm away from the posterior wall of the left atrium. **b** A 12-year-old boy with a parahisian accessory pathway ablated with a cryoablation catheter positioned in the His region. **c** A 13-year-old boy with right posterolateral focal atrial tachycardia with the ablation catheter in contact with the myocardium during radiofrequency (RF) ablation. The yellow circle shows myocardial echogenicity at the beginning of RF application. **d** The myocardium in contact with the RF catheter suddenly becomes brighter and larger, corresponding to the oedema caused by RF application

One of the primary advantages of ICE is its ability to deliver optimal visualization of cardiac structures and catheter placements during ablation procedures. Unlike traditional imaging techniques, ICE provides high-resolution images of the heart’s anatomy in real time, which is crucial when navigating the complex and often delicate structures of the paediatric heart. This enhanced visualization not only aids in the accurate positioning of catheters but also allows for the identification of anatomical variations that may otherwise pose challenges during the procedure. This rapid acquisition of data enhances the procedural approach by minimizing time spent under fluoroscopy, ultimately contributing to improved outcomes. This is particularly important in paediatric patients when performing transeptal punctures where the size of the left atrium is smaller compared to adults, and often the tip of the transeptal needle is at risk of piercing the posterolateral wall of the left atrium ([17]; Fig. 4a). The use of ICE has also been beneficial in paediatric AVNRT patients to precisely localize the region of the slow pathway [18] or parahisian accessory pathways, where the risk of AV block is higher (Fig. 4b). Its use is also paramount in patients with complex congenital heart disease, where fluoroscopy will not be sufficient to identify critical cardiac structures, and 3D mapping reconstructions only provide still anatomical reconstructions [19–21].

Furthermore, the real-time imaging capabilities of ICE profoundly influence clinical decision-making during catheter ablation. Based on the instantaneous feedback on catheter positioning and cardiac structures, electrophysiologists can immediately adjust procedural decisions in real time, as critical information is visualized. Using ICE not only provides the interventionist with valid information on the positioning of the catheter in relation to anatomical structures but also offers real-time critical information of dynamic tissue alterations during RF applications. The echogenicity of such tissue alterations can be visualized using ICE, and with this comes the opportunity for the “live” visualization of lesion generation/expansion within the targeted tissue, and additionally the potential, for example, to avoid steam pops by recognizing specific harbingers of such events (Fig. 4c, d). This opportunity for dynamic decision-making during RF applications reduces the likelihood of complications associated with catheter misplacements or inadequate ablation. It is particularly beneficial in managing complex arrhythmias, where the flexibility to rapidly alter strategies can lead to higher success rates. Hence, ICE not only enhances the initial procedure but also informs follow-up strategies based on real-time data.

Additionally, integrating ICE with other imaging modalities enhances its overall utility. Real-time fusion techniques that merge ICE with 3D mapping systems afford a comprehensive view of both anatomy and electrical activity during catheter ablation. Currently, only Biosense Webster’s CartoSound (Diamond Bar, CA, USA) [22] offers this option, but Abbott has announced that it will be available in new versions of its EnsiteX. This multifaceted approach ensures high accuracy in identifying target areas for ablation while reducing the time spent under fluoroscopy—a crucial factor in paediatric settings, particularly those involving complex congenital heart diseases where minimizing radiation exposure is paramount.

The benefits of ICE extend beyond immediate procedural outcomes. By fostering a safer environment during catheter interventions, ICE has the potential to improve the long-term health of paediatric patients. Ensuring that these interventions are conducted with minimal radiation exposure can lead to lower incidence rates of radiation-induced complications over time. The emphasis on patient safety, especially in a vulnerable population, is fundamental to the evolving practices in paediatric electrophysiology. The role of ICE, therefore, is not merely as a supplementary tool but also as a cornerstone for advancing the paradigms of care within this realm.

In summary, the incorporation of ICE into paediatric catheter ablation procedures represents a transformative development in clinical practice. Its capacity to enhance procedural outcomes by improving visualization, facilitating dynamic clinical decision-making, and reducing reliance on fluoroscopy addresses essential safety concerns. As the field of electrophysiology continues to evolve, the integration of ICE represents a critical step towards delivering high-quality, safe, and effective care for paediatric patients suffering from arrhythmias. Ongoing rigorous research and implementation of innovative imaging modalities such as ICE will play a vital role in shaping the future of paediatric electrophysiology.

## Utilizing non-invasive digital twins

The advent of non-invasive digital twin technology offers unprecedented potential for the treatment of paediatric patients with cardiac arrythmias, particularly in terms of refining both anatomical understanding and procedural planning. Digital twins, which are virtual representations of a patient’s unique anatomical and electrical configuration derived from data collected through advanced imaging modalities such as cardiac magnetic resonance (MRI), cardiac computed tomography (CT), echocardiography, and ECG, can significantly enhance pre-procedural insights tailored to individual cases. The development of cardiovascular digital twin frameworks that meaningfully impact medical practice requires accurate patient representation based on diverse data types while maintaining continuous updates through sensor data streams. Digital twins must mimic underlying physiological systems and organs by capturing the body’s hierarchical structure from cells and tissues to organs and organ systems [23]. The goal is to capture this hierarchical structure to enable hierarchical design and composition with sufficient personalization at multiple levels. However, the feasibility of modelling individual physiology starting at the molecular level remains questionable due to both theoretical and practical challenges. A complete digital twin starting at the cellular level would require complex quantum biochemical simulations that overcomplicate practical applications without reasonable justification. Therefore, the level of resolution and personalization should be driven by clinical applications, with the motivation being to provide maximum utility rather than achieve theoretical completeness [24].

**Fig. 5:**
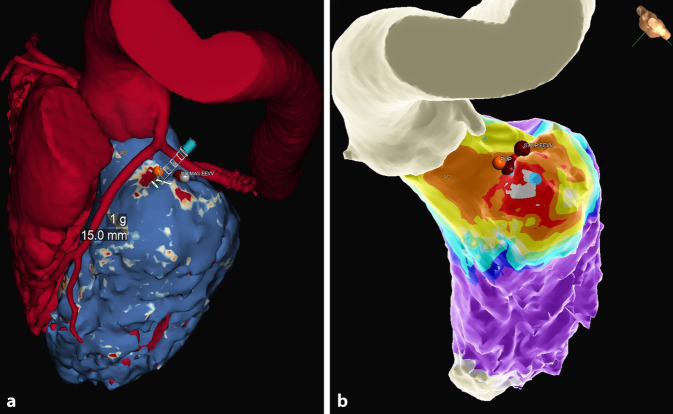
A 14-year-old boy with ventricular tachycardia originating from the left ventricular summit. **a** Cardiac computed tomography (CT) scan of the patient to identify coronary arteries and analyzed with ADAS 3D software to identify regions of possible fibrosis. **b** Ensite X local activation time map integrated with the ADAS 3D CT scan. Note how the earliest activation region corresponds to regions of possible fibrosis identified with ADAS 3D. Successful ablation points shown on the map

Focusing on clinical applications that can be, and already are, used in day-to-day practice, 3D mapping systems and cardiac MRI using late gadolinium enhancement identification are two good examples of patient-specific models that help with the identification and characterization of a patient’s arrhythmogenic substrate. Moreover, new tools have been developed to combine this information in a single solution. ADAS 3D is novel software that can semiautomatically quantify myocardial scar using CT or MRI and successfully identify possible electrical cardiac corridors within the scar tissue that are the arrhythmogenic substrate for ventricular arrythmias (Fig. 5a, b). This novel technology has been validated in the Post-Ablation cardiac Magnetic resonance to assess Ventricular Tachycardia recurrence (PAM-VT) study for VT ablation in adults. ADAS 3D has been shown to help in identifying the substrate for VTs and in planning for their ablation. It was also successfully applied for the prediction of a long-term absence of VT recurrences when the absence of preprocedural cardiac corridors was demonstrated on a post-ablation MRI or CT scan [25]. At present, this novel technology has been validated for ischemic VT ablation, but in the future, it may prove extremely useful for patients with congenital heart diseases, in whom most of the atrial tachycardias or VTs are related to myocardial scars [26]. Its application will be particularly useful for adult patients after surgery, such as tetralogy of Fallot patients, in whom non-invasive identification of critical slow-conducting ventricular isthmuses can be performed in order to individualize the ablation strategy and define which arrhythmogenic substrate will be targeted [27]. It may also be useful for estimating the risk of developing potentially prognostically relevant arrhythmias, thereby guiding decision-making for preventive ablation or device implantation.

**Fig. 6:**
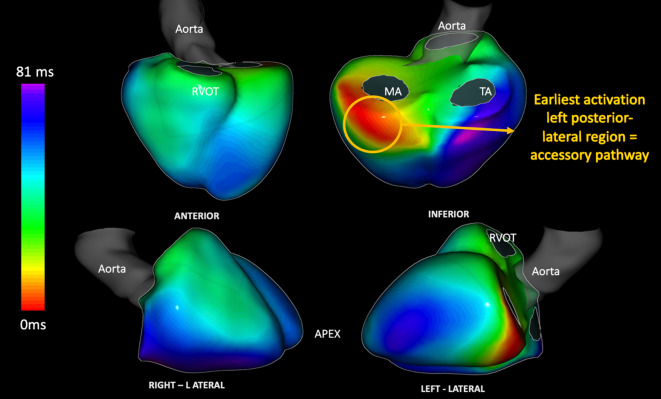
A 12-year-old boy with a left lateral accessory pathway non-invasively identified with the ACORYS electrocardiographic imaging system prior to radiofrequency ablation. *RVOT* right ventricular outflow tract, *MA* mitral annulus, *TA* tricuspid annulus

Another emerging cardiac digital twin technology that will revolutionize the treatment of paediatric arrythmia is electrocardiographic imaging (ECGI). This technology uses a body-surface ECG vest to non-invasively represent electrical anatomical mapping (CardioInsight vest [Medtronic, Minneapolis, MN, USA] or ACORYS® [Corify Care, Madrid, Spain]) [28] and help visualize electrical wave propagation. It has been successfully used to plan and predict ventricular synchrony in patients with different types of cardiac resynchronization therapy or physiological pacing [28, 29], but it has also been used to non-invasively identify the origin of premature ventricular complexes [30, 31] and VT [32]. Recent advances in this technology have been used in children with other arrhythmic substrates, and ECGI can successfully predict the precise localization of accessory pathways during both sinus rhythm and orthodromic tachycardia (Fig. 6).

In summary, the incorporation of non-invasive digital twins into paediatric catheter ablation signifies a transformative leap in the field of electrophysiology. The technology’s potential to refine anatomical understanding, optimize procedural planning, reduce radiation exposure, and support personalized treatment strategies sets the stage for safer and more effective interventions.

## Focal pulsed field ablation catheters

Pulsed field ablation (PFA) has become increasingly relevant in the field of cardiac electrophysiology. In contrast to thermal lesion formation using CRYO or RF ablation, PFA lesions are created by non-thermal irreversible electroporation, which is considered to be more selective for cardiac tissue. PFA is a largely non-thermal energy approach that involves the use of microsecond-scale, high-voltage electrical fields to cause irreversible electroporation and destabilization of cell membranes, a process that culminates in cellular necrosis. Preclinical and clinical studies have shown that PFA has a degree of ablative specificity that allows myocardial tissue to be preferentially ablated with limited effects on adjacent tissues such as the oesophagus, phrenic nerve, and pulmonary vein tissue, and it has been shown to be safe and effective for the treatment of atrial fibrillation in adults using circumferential multipolar catheters [33]. As this technology is evolving, specific PFA generators (CENTAURI™ System, Galvanize Therapeutics, Inc., Redwood City, CA, USA) can be used with specific PFA catheters. Systems of this kind can even be used with conventional RF contact force (CF) cardiac ablation catheters and 3D mapping systems (EnSite™/TactiCath™ SE [Abbott, Abbott Park, IL, USA], Carto® 3/ThermoCool SmartTouch™ [Irvine, CA, USA], Rhythmia HDx™/IntellaNav Stablepoint™ [Boston Scientific, Burlington, MA, USA]) that are currently commonly used for RF ablation or CRYO in the treatment of paediatric and congenital cardiac arrythmia substrates such as AVNRT, AVRT, FAT, macroreentrant atrial tachycardia, and VT [34].

RF and CRYO energy sources are widely used and remain the basis of safe and effective treatment tools for cardiac ablation in children, with success rates of approximately 87–99%. The effective application of RF depends on key procedural factors, including type of energy, power output, application duration, catheter–tissue CF, and stability during energy delivery [35]. Achieving consistent catheter stability and moderate CF can be particularly challenging in anatomically complex regions, such as the superior tricuspid annulus (TA) or lateral mitral annulus (MA). To compensate for this, clinicians often increase power settings to enhance tissue heating and lesion formation through thermal conduction. However, prolonged high-power applications carry the risk of serious complications, such as AV conduction block, coronary artery damage, and cardiac perforation due to uncontrollable deep tissue steam formation, leading to ruptures of the surrounding tissue (steam pop). These limitations highlight the need for strategies offering the chance to optimize both safety and efficacy in accessory pathway ablation. PFA is evolving into a promising alternative to RF ablation by addressing several of its shortcomings. In contrast, PFA creates transmural and contiguous lesions with less reliance on consistent catheter–tissue CF, simplifying procedures in challenging anatomical areas. Its unique method of energy delivery is able to avoid damaging different tissues; therefore, it might be of interest in reducing the risk of damaging the His Bundle, combining safety and efficacy. PFA has been shown to be safe and effective in ablating accessory pathways [36], AVNRT [37], complex FAT in adults [38], as well as in a few paediatric cases [39, 40]. One of the main drawbacks of using PFA lays in the risk of coronary artery spasm [41] while ablating substrates in close proximity to the AV junction and coronary arteries, which could be prevented recently with the infusion of intravenous nitrates. Another potential risk is transient sinus arrest during PFA, but this recovered spontaneously [36].

## Practical conclusion

The advances in paediatric catheter ablation techniques represent a significant step towards enhancing the safety and efficacy of interventions in young patients. The integration of innovative imaging systems and mapping technologies has been instrumental in minimizing radiation exposure, which is of critical concern in the paediatric population. Techniques such as intracardiac echocardiography, novel mapping algorithms, and non-invasive digital twins enhance procedural planning and precision while ensuring high success rates with reduced complications. The emerging focal pulsed field ablation catheters might further improve patient outcomes by preserving essential cardiac structures during interventions.

## Data Availability

All figures have been anonymized, and it is impossible to identify the owner.

## Declarations

Ethics approval and consent to participate: Since this is an editorial review, no patient data were used to write this article, and therefore no ethic’s approval was needed. All figures have been anonymized.
